# Balance in life as a prerequisite for community-dwelling older adults’ sense of health and well-being after retirement: an interview-based study

**DOI:** 10.1080/17482631.2021.1984376

**Published:** 2021-10-11

**Authors:** Catharina Gillsjö, Maria Nyström, Lina Palmér, Gunilla Carlsson, Ann-Charlotte Dalheim-Englund, Irene Eriksson

**Affiliations:** a School of Health Sciences, University of Skövde, Skövde, Sweden; b Faculty of Caring Science, Work Life and Social Welfare, Department of Caring Science, University of Borås, Sweden; c College of Nursing, University of Rhode Island, USA

**Keywords:** Health, lifestyle, older adults, community, life balance, retirement, well-being, qualitative, phenomenography

## Abstract

**Purpose:**

This study aimed to describe community-dwelling older adults’ perceptions of health and well-being in life after retirement.

**Methods:**

This study is part of a larger project using a mixed-methods design to address lifestyles’ influence on community-dwelling older adults’ health. Individual semi-structured interviews were conducted with 18 older adults in age 70 to 95 years. Data were analysed according to a phenomenographic approach.

**Results:**

The results encompass four categories describing variations in community-dwelling older adults’ perceptions of health and well-being after retirement: *feeling well despite illness and disease, interacting with and being useful for oneself and others, independently embracing opportunities and engaging in life*, and *maintaining a healthy lifestyle.*

**Conclusions:**

The absence of illness and disease is not a clear prerequisite for a sense of health and well-being. To promote and preserve health and well-being after retirement, older adults strived for—and coached themselves to uphold—a balance in life, focusing on not burdening others. This life orientation after retirement must be acknowledged by society at large, especially from an ageist perspective, and in health and social care to preserve and promote health and well-being.

## Introduction

Older adults are a fast-growing age group both worldwide and in Sweden. In Sweden, and according to the World Health Organization (WHO) (WHO, 2013), *older adults* are commonly defined as individuals aged 65 years and older. Sweden’s population stands at approximately 10 million people, 2 million of whom (20%) are aged 65 years or older. This age group is expected to increase over the coming decades, and average lifespans are expected to increase simultaneously. Ageing affects the human body biologically, psychologically, and socially, but the rapidity of the ageing process varies by individual. It also varies depending on one’s health problems and the extent to which they affect an individual’s daily life.

### Ageing’s impact on older adults’ lifestyles

Ageing implies becoming more vulnerable to chronic diseases, which may lead to dependency and a greater need for care; therefore, older adults’ health conditions are closely related to functionality, including independence in daily life, and autonomy (Feng et al., 2017). Although ageing is often associated with impaired functions, diseases, and losses, being old can still have positive aspects. Good ageing can be described by concepts such as *healthy ageing, active ageing*, and *successful ageing*, including physical, psychological, social, and spiritual dimensions (Nygren, 2006). The present study focuses mainly on the concept of healthy ageing. The WHO (WHO, 2019) defined *healthy ageing* as “a process of maintaining functional ability to enable well-being in older age.” Thus, some important components of functional ability include building and maintaining relationships, as well as contributing to society. A central obstacle to healthy ageing that research has highlighted is the pervasive stereotypes and negative attitudes towards older adults among society at large and especially among healthcare providers. The ageist perspective that prevails among healthcare providers affects older adults’ sense of health and well-being, resulting in an increased risk of negative thoughts, social isolation, depression, and shortened lifespans (Burnes et al., 2019; Officer et al., 2016; WHO, 2019; Wilson et al., 2017; Wyman et al., 2018).

Despite a great impact on daily life, evidence suggests that the majority of community-dwelling older adults prefer to remain in their own homes as they age since homes are often closely linked to older adults’ identities, integrity, and lifestyles (Gillsjö, 2012; Wiles et al., 2012). Maintaining relationships can be difficult for many older adults who experience loneliness and social isolation (Courtin & Knapp, 2015). Loneliness comprises several varieties—such as social, emotional, and existential (Bolmsjö et al., 2019; Weiss, 1973)—all of which can become problematic after retirement. Researchers have sometimes used the term *loneliness* to reflect these three varieties, sometimes distinguishing between social, emotional, and existential loneliness. Loneliness and social isolation are common problems among older adults, and they have been associated with an increased risk of health problems and changes to quality of life, emphasizing the need for increased attention to these problems in research and clinical practice (National Academies of Sciences Engineering and Medicine, 2020; WHO, 2021) *Social loneliness*, can be described as the discrepancy between a person’s chosen and actual levels of social contact, and it involves a subjective dimension; meanwhile, social isolation involves an objective perspective and is described as having minimal social contact with other individuals (Peplau & Perlman, 1982). These two concepts are sometimes considered synonymous with *living alone*, but although they are related, these concepts do not overlap (Wenger et al., 1996). Research has shown that living alone does not necessarily indicate loneliness among older adults, many of whom have reported frequent social contact and active social involvement in community organizations despite living alone. However, evidence (Steptoe et al., 2013) suggests that experiences of loneliness or social isolation are a major risk factor for physical and mental illness later in life. Reports have also shown that people with large, active social networks live longer and in better health, describing higher levels of well-being than people who lack such networks. Improvements in quality of life, functionality, mortality, and subjective well-being have also been described (Amati et al., 2018; Avlund et al., 2004; Bruine de Bruin et al., 2020; Lund et al., 2000; Valtorta et al., 2016).

### Previous literature

Various concepts—such as quality of life, life satisfaction, morale, and psychological or subjective well-being—have often been used synonymously in the literature (Ranzijn & Luszcz, 2000). Since *well-being* is associated with a variety of physical health conditions and is perceived as a public health priority, it must improve among older adults (Fancourt & Steptoe, 2018). Several authors have tried to define the concept using objective conditions, but such attempts have been criticized because a standard is lacking and only older adults can decide whether they perceive well-being as a condition that has affected their life and sense of well-being (Boggatz, 2016). Physical and psychological health can be affected by passive lifestyles and also influence subjective well-being (Chui, 2018; Djernes, 2006; Kikuchi et al., 2014; Lampinen et al., 2006; Warburton et al., 2006). Previous research has associated a meaningful life with a sense of health, as well as well-being (Hedberg et al., 2010; Ju, 2017; Steptoe et al., 2013). After retirement, maintaining an active lifestyle and a meaningful life is even more important. During transitions to retirement, a shift in daily life can often imply opportunities to promote active lifestyles, potentially facilitating healthy ageing (Ter Hoeve et al., 2020). Physical activities are well known to greatly influence health and well-being, and regular exercise can extend lifespans by reducing the risk of developing lifestyle-related diseases (Warburton et al., 2006).

Another important resource in promoting and preserving well-being among older adults is inner strength Lundman, Aléx (Lundman et al., 2012), which is a complex phenomenon (Viglund et al., 2017). Lundman, Aléx (Lundman et al., 2012) also suggested that a central component of promoting health and supporting healthy ageing is a salutogenic approach, focusing on older adults’ strengths and capacities—not only on their weakness and frailty. The theoretical model Lundman, Aléx (Lundman et al., 2010) described is based on four interacting dimensions: *connectedness, creativity, firmness*, and *flexibility. Inner strength* means an ability to view various changes as a natural part of life. It also means being open to living conditions and managing challenges and opportunities. Additionally, it entails seeing life as meaningful and worthy of engagement, as well as being creative and flexible and making life choices with an orientation that contributes to meaning in life. Inner strength has also been linked to factors such as family, friends, society, nature, and spirituality, and it is affected in various ways by life events (Chui, 2018).

Previous research (Dalheim-Englund et al., 2020; Palmér et al., 2020, 2020) focusing on healthy older adults’ lives after retirement and the meaning of ageing have shown that post-retirement freedom is highly valued. Moreover, this period exposes older adults to an increased risk of illness and disease. Research has shown that questions and thoughts about meaningfulness occur when one approaches the end of their life. Ageing science (Laslett, 1991, 1994) has shown that, for people aged 65 years and older, the ageing process comprises various phases, termed the *third* and *fourth age*. These stages are related, rather than bound, to chronological, biological, psychological, and social age. The fourth age is characterized by impaired function and well-being as a result of illness and symptoms, and the transition to the fourth age usually occurs gradually, with an increasing disability that negatively affects health and well-being. The third age occurs alongside retirement and is characterized by good health and function, activity, participation, and well-being.

However, limited research has focused on community-dwelling older adults in their third age and examined life after retirement. It is, therefore, important to explore older adults’ perceptions of their health and well-being after retirement. Accordingly, the current study sought to address this limitation.

### Aim

This study aimed to describe community-dwelling older adults’ perceptions of health and well-being after retirement.

## Materials and methods

### Design

This study is part of a larger project using a mixed-methods design to address lifestyles’ influence on community-dwelling older adults’ health (Dalheim-Englund et al., 2020; Gillsjö et al., 2021; Palmér et al., 2020, 2020). The project as a whole comprises quantitative data collected through a lifestyle questionnaire and a genetic analysis of saliva samples, as well as qualitative, semi-structured interviews. The questionnaire comprised closed- and open-ended questions regarding demographic data, medical and other health-related histories, social relationships, health self-evaluations, post-retirement lifestyle changes, and meaning in life. Three validated questionnaires were included in this study’s questionnaire: “Sense of Coherence—13” (SOC-13), “Sense of Coherence-Emotional” (SOC-E), and “Sense of Meaning Profile” (SOMP) (Antonovsky, 1993; Antonovsky et al., 1991; Flensborg-Madsen et al., 2006; Langius & Björvell, 1993; Reker, 1996). Profile groups were created and compared statistically, based on questionnaire responses, to provide a basis on which to ask older adults to participate in the current study’s interviews. Information regarding questionnaire scores was not used in the study’s data collection or analysis phases since our strategic sampling aimed to capture a wide range of phenomena. A phenomenographic approach derived from Marton’s work at the pedagogical institution at Gothenburg´s University (Marton, 1981, 1986; Marton & Booth, 1997) was chosen to describe the variation in older adults´ perceptions of qualitatively different ways to perceive the phenomena, community-dwelling older adults’ perceptions of health and well-being after retirement.

### Setting and participants

The prospective persons invited to participate in this study were community-dwelling older adults aged 70–95 years who considered themselves healthy. Their recruitment was conducted in collaboration with the Swedish association Active Seniors in the south and middle parts of Sweden. Older adults were invited to participate in a seminar with the theme of “genes and healthy aging” through an advertisement in local newspapers and through Active Seniors. At the seminar, prospective participants were informed orally and in writing about the project. Attendees who consented to participate answered the lifestyle questionnaire and provided a saliva sample.

Attendees were also asked if they wished to participate in an interview. Those who agreed consented and provided their telephone number in the same document as their consent to participate in the lifestyle questionnaire and saliva sample parts of the study.

A strategic sample was drawn from profile groups of participants that were created based on responses to the lifestyle questionnaire in order to achieve a wide variety of participants’ backgrounds and experiences. Combinations of high and low scores in participants’ responses across the SOC-13, SOC-E, and SOMP (Antonovsky, 1993; Antonovsky et al., 1991; Flensborg-Madsen et al., 2006; Langius & Björvell, 1993; Reker, 1996) questionnaires were used to achieve this variety, together with gender and age as the basis for the study’s strategic sampling.

Twenty-three seniors were called on the telephone and asked if they still agreed to participate in the study’s interview portion. Six of these respondents had changed their minds, so 18 older adults (eight men and 10 women) aged 72 to 91 years (M = 78) took part in the interview study. Twelve participants were married or living with a partner, and six were living alone. All participants were living in ordinary housing, without healthcare or social care. All but one of the 18 participants perceived their health statuses as *very good* or *satisfactory*.

### Data collection

Individual semi-structured interviews were conducted at participants’ homes or in other settings, as decided by each participant, in order to enable an understanding of the phenomenon of community-dwelling older adults’ perceptions of their health and well-being after retirement. The interviews were conducted by researchers experienced in conducting qualitative interviews with older adults. An interview guide was developed and used to guide the interviews, in which participants were asked to describe their experiences of health and well-being, healthy ageing, methods of preserving and promoting health and well-being, lifestyles, post-retirement changes, and thoughts about meaning in life. A lifeworld approach was used in the interviews, focusing on the world as it is experienced by respondents (Dahlberg et al., 2008).

The interviews’ opening question was, “Can you please tell me what good health is to you?” The subsequent questions were:

- “What is most important in your daily life?”
- “What do you do to ‘feel good’?”
- “What does it mean to be healthy in old age?”
- “What changes have you made in life since retirement?”
- “What gives you meaning in life?”
- “What do you think about the future?”

Follow-up questions were asked to probe responses, obtain more detailed answers, and further encourage reflection in order to reveal underlying meanings and descriptions of interviewees’ feelings and thoughts on their post-retirement life. These probing questions helped respondents repeat, clarify, elaborate, and confirm their interview answers about how they perceived the studied phenomena. The interviews were audio-recorded and transcribed verbatim.

### Data analysis

The study’s data analysis was conducted according to the phenomenographic approach created by Dahlgren and Fallsberg (Dahlgren & Fallsberg, 1991) and further developed by Sjöström and Dahlgren (Sjöström & Dahlgren, 2002) for use in nursing-science phenomenographic analyses. This approach comprises seven steps undertaken iteratively, repeating steps as necessary and progressing through the steps in any order, rather than a linear process. The researchers’ prejudices and preunderstandings of the studied phenomena were restrained in the processes of data collection and analysis to reveal how participants understood the studied phenomena (Sandbergh, 1997).

During the first step, *familiarization*, the researchers became closely acquainted with the study’s textual data through reading each interview transcript several times. Five of the six authors conducted the interviews, facilitating the familiarization step, and all six authors read the transcripts to become familiar with the content. The first and last authors then conducted the analysis. The next step, *compilation*, involved identifying and compiling the content of each informants´ answers regarding the phenomenon, community-dwelling older adults’ perceptions of health and well-being after retirement. The third step, c*ondensation*, involved reducing these compilations by identifying the central parts, the core content in the informants´ answers. The fourth step, *grouping*, entailed classification and grouping similar responses. Six groups were distinguished to reflect variations in participants’ perceptions of their health and well-being after retirement. These groups constituted the study’s preliminary categories. The fifth step, *comparison*, entailed establishing and confirming the borders between these preliminary categories. During this step, the number of categories was revised, and four of the preliminary categories were fused into two categories for qualitative distinction. During the sixth step, *naming*, each of the four remaining categories was labelled to linguistically reflect their respective cores. Then, during the seventh and final step, *contrastive comparison*, each of the four categories was described to reflect its unique character. The final categories describing the studied phenomena constituted an *outcome space*—a structural framework in which descriptive categories exist and relate to each other (Marton, 1981, 1986; Marton & Booth, 1997).

The interview transcripts’ reliability in the analysis process was considered since all authors had read the transcripts to become familiar with their content. This consideration took place after the researcher responsible for conducting the interviews had read the transcript text, clarifying passages through which respondents had been difficult to hear and checking for typing errors. The credibility of the analysis process was also considered in that the authors had conducted the interviews, which served as a useful step in familiarizing them with the transcripts’ content and relation to the study’s purpose. This credibility was further enhanced during the detailed description of the steps in the analysis process and the researchers’ awareness of their pre-understandings (Marton, 1986; Sandbergh, 1997; Sjöström & Dahlgren, 2002; Stenfors-Hayes et al., 2013). The study’s data analysis was conducted by the first and last authors through a close-reading, phenomenographical approach. These authors discussed the preliminary categories several times in relation to the empirical data, reaching a consensus regarding the data analysis, confirming the borders between the identified categories, and labelling the descriptive categories in the outcome space and confirming their relation to one another. This process—called “negotiated consensus” by Wahlström and Dahlgren (Wahlström et al., 1997)—was used to enhance the credibility of the current study’s analysis. Interjudgereliability and intersubjective agreement were considered and achieved since the co-authors identified description categories in the interview excerpts and interview transcripts as a whole (Marton, 1986; Sandbergh, 1997; Stenfors-Hayes et al., 2013).

### Ethical considerations

This study was conducted following national ethical regulations, and it conforms to the *Declaration of Helsinki* (Declaration of Helsinki, 2013). Participants were informed about the study, both verbally and in writing, before providing informed consent to the study’s aim and data collection procedure, data confidentiality and pseudonymization (including the protection of participants’ identities in the study’s analysis), and publication. They were also informed that their participation was voluntary and that they had a right to withdraw from the study at any time and without consequences. The Regional Ethical Review Board approved the whole project of which this study is one part (Dnr. 983–13).

## Results

This study’s outcome space comprised four categories describing variations in the phenomenon of community-dwelling older adults’ perceptions of their health and well-being after retirement: *feeling well despite illness and disease, interacting with and being useful for oneself and others, independently embracing opportunities and engaging in life*, and *maintaining a healthy lifestyle*. Each category was described and illustrated with excerpts from the interviews with participants.

Theoretically, the outcome space put the internal relations between description categories in perspective, revealing variations in older adults’ requisite elements for a sense of health and well-being. The internal relations between categories were both hierarchical and linear. A hierarchical relationship occurred with the category *feeling well despite illness and disease* predominating and prevailing throughout the other categories as a throughline and ultimate goal. The other description categories can be understood as both perceptions of the studied phenomenon and ways to reach the sense of feeling well despite illness and disease.

### Feeling well despite illness and disease

Health and well-being were perceived as encompassing feeling well despite illness and disease. Even if the older adults wished for good health in terms of being free of illness and disease, they expressed also understanding *good health* in the sense of being free of illness and disease as unnecessary for feeling well. They felt “a need to feel well” and expressed that it was “most important to feel well psychologically to feel well and to function physically.” The older adults expressed that “one knows nothing about what is going to happen … nothing can be taken for granted.” Although, they had the orientation to “try not to worry and take the day as it comes” to uphold a sense of health and well-being. Feelings of sadness and anxiety were present and associated with ageing and related health problems. There was also uncertainty and fear related to not being prioritized and have accessibility to healthcare when needed “I worry and it’s unfair not get the healthcare needed due to high age.” Despite anxiety and fears related to illness and disease, they strived for a sense of health and well-being in daily life. The older adults valued and had a wish to wake up at home in the morning, feeling healthy, with their body feeling good, alert, vital, and free of pain. The sense of well-being was described as “a feeling of good health in body and soul.” The older adults expressed an awareness that one could have a disease without knowing, but they also explained that they could feel a sense of well-being as long as they felt healthy in body and soul. They suggested that one can live a good life and feel well despite illness and disease. Moreover, they explained that the ability to feel healthy and remain active sustained the feeling that “one forgets that the body is old; I still feel that I’m 50,” and such perspectives added to their sense of feeling well. They also noted that taking time to reflect and have the ability to view the current situation and health problems from the bright side and to feel satisfied, played a significant role in preserving their overall sense of well-being.

### Interacting with and being useful for oneself and others

The older adults highlighted their own responsibilities in their interactions and to achieve their own well-being. They valued opportunities to decide how to spend their time, and they balanced their time between being alone and interacting or socializing with others. The older adults expressed that “time alone is important to feel well” but, at the same time, highlighted the importance of “not feeling alone involuntarily.” They prioritized togetherness and creating a good home life through balanced interactions with the self and others in order to feel well. The importance of interacting with others to feel well could be understood through such remarks as, “It’s frightening to think of being left alone.” A need to be useful, requested, appreciated, and confirmed was noted as a component of feeling significant, part of a sense of well-being. They also strove to uphold “good relationships that make you feel well” and tried to avoid “unhealthy” relationships, including tensions and conflicts.

Older adults highly appreciated spending time with their family members and friends, since this contact promoted and preserved their sense of well-being. Although they highly valued spending time with their family members, they expressed caution towards their interactions with their families, such as visiting their children and grandchildren. They expressed an awareness that they should not rely too much on their children in order to avoid becoming a burden, since they knew such dependency would make them feel unwell. Their dialogues with their children were important since participants associated their children’s well-being with their own well-being. The older adults also valued active relationships and spending time with friends who shared their interests, such as participating in associations. Moreover, respondents also highlighted the significance of taking time for oneself to reflect or do solo activities to promote their overall well-being.

Older adults expressed an awareness of the need to make the most of their own lives based on experiencing friends’ health problems or feeling unwell. Honesty towards oneself and others, “doing the right thing” meaning acting in accordance with one’s principles and values, maintaining a clear conscience, and helping others were noted as significant aspects of their interactions with themselves and others. They associated a sense of well-being with being useful and with the validation of one’s efforts: “It’s needed to feel good in the soul.” Supporting and helping others were expressed as significant components of sustaining one’s own well-being—especially encouraging and helping others who felt unwell. All interactions with pets were also explained as a source of well-being, especially when living alone.

### Independently embracing opportunities and engaging in life

Older adults valued the freedom and opportunities to care for themselves and make their own decisions in their daily lives, noting that “freedom is important for health and well-being … to decide for oneself” and also significant to “take on life positively and focus on opportunities in life.” Retirement was described as a relief, allowing own choices about how to spend their days. They emphasized the importance of consciously choosing a lifestyle that suited them and allowed them to feel well. The freedom to independently deal with and manage their daily lives was also highly valued. Moreover, older adults expressed a desire to remain at home, control their daily lives, and make their own decisions. They expressed the significance of embracing each moment: “I’m happy to wake up every morning, to get out of the bed and make breakfast” and live in the present. The older adults also cherished their ability to independently engage with and embrace life in order to remain in good health and feel well. They highly valued the ability to make their own decisions about how to spend their days, including planning their meals and being able to “carry out daily inside and outside chores without help.” Their highly valued activities included engaging in life and taking time to “enjoy spending time in the forest or in the garden.” Financial situations were highlighted as an important aspect of remaining free and independent since the “economy is important; thus, it means quite a lot.” Older adults embraced opportunities to stimulate their cognitive abilities and felt a “need to do things to keep the brain going” in order to maintain their health and well-being.

Happiness was expressed as a result of feeling well, and the older adults explained that their major focus to sustain that feeling was things that had contributed to joy and meaning in life. For example, one interviewee stated, “Meaningful activities facilitate good health and well-being.” Engaging in interests and pursuing creative activities—such as painting, dancing, or learning a new language—were noted as ways to promote and preserve their sense of well-being. Life was perceived as easier when one felt well compared to when one felt unwell, and the older adults expressed facing life somewhat courageously. They explained, “Everything goes easy and smoothly when focusing on doing things that make you happy. It’s rather easy to distance boring thoughts.”

Some older adults described avoiding activities that reminded them of life’s finality since they did not want to die, stating that “the death and I do not get along” and, therefore, that they avoided activities related to death as “death cleaning.” Others were unafraid of death and had faith in God and the afterlife, which contributed to a sense of meaningfulness and safety in their lives. They emphasized that allowing themselves to enjoy life without a bad conscience “makes it rather easy to distance boring thoughts.” They also stressed the importance of creating a meaningful life as a component of sustaining a sense of well-being since “activities are needed; otherwise, life becomes boring.”

The older adults consciously focused on well-being and strove to feel well in life, doing the best they could in their respective situations. They embraced and engaged in life since they wanted to live and participate in life for as long as possible. In addition to engaging in their existing interests, they did not hesitate to learn about new interests. They expressed an awareness of the need to balance their independence—embracing opportunities and engagements—with avoiding pressuring themselves in life. This conscious balancing aimed to avoid situations in which one felt unwell.

### Maintaining a healthy lifestyle

The older adults also emphasized the importance of maintaining a healthy lifestyle, balancing rest with activities in order to feel well. They described the importance of consciously choosing a lifestyle, as well as striving towards and coaching themselves in a healthy lifestyle. Moreover, they valued the strength to be active and to invest the necessary time to sustain and challenge their physical and psychological abilities, promoting and upholding a sense of feeling well. The older adults also highlighted the importance of taking advantage of good days—remarking, for instance, “The health is a little up and down. … When I’m free of pain, I take a walk or go fishing.” They also stressed the importance of living a healthy life despite illness and disease in order to feel well. This drive was expressed through such remarks as, ”I don’t want to lie down and be lazy. … It’s a part of my world to be in motion every day in spite of difficulties to walk, to walk in the forest or around the lake, four kilometres three times a week.”

Maintaining a healthy lifestyle and staying active helped the older adults avoid uncomfortable thoughts and feelings, improving their sense of well-being. They described the importance of keeping up habits and routines in order to feel well and remain active—for example, getting out of bed every morning, managing personal hygiene, eating and sleeping well, and carrying out activities. Physical activities—such as working full days, spending time in nature, taking walks, pursuing carpentry, gardening, and training at the gym—were undertaken to maintain a healthy lifestyle as help them feel well. Participating older adults also expressed the “theory of doing something every day that have to do with physical activity.” The importance of daily physical activities that raised heart rates was emphatically noted. An effort to “raise the heartbeat above 100 and to sweat at least one time a day” was highlighted as a way to maintain a healthy lifestyle and feel well.

The older adults also stated that ”nutrition is important, nutritious and varied nutrition,” as a key to a healthy lifestyle, including limited alcohol use. Mental stimulation and cognitive challenges were noted as significant aspects of remaining healthy and upholding a sense of well-being. Related activities included crosswords, listening to music, art, using a computer, and developing or creating things. Keeping occupied was recurrently noted as an aspect of promoting and preserving one’s sense of well-being.

## Discussion

This study aimed to describe phenomenological variations in community-dwelling older adults’ perceptions of health and well-being after retirement. The study’s results revealed the centrality of feeling well despite illness and disease and the importance of a balance between (a) interactions with the self and others and (b) independently embracing opportunities, and (c) engaging in life and maintaining a healthy lifestyle in order to feel well. The older adults expressed a need to feel useful, requested, appreciated, and confirmed as important in order to maintain a sense of well-being.

Health and well-being were perceived as feeling well even despite illness and disease. Feeling well despite illness and disease was the most dominant aspect throughout the study’s results regarding older adults’ perceptions of their health and well-being. Remaining healthy was an overall desire among the older adults. The importance of feeling well in body and soul and of waking up every morning feeling healthy, vital, and mentally and physically alert—as well as free of pain—was a recurring wish at the forefront of the study’s interviews. Ageing has been associated with increased vulnerability, illness, and disease, and it often accompanies a functional or psychological decline that contributes to a subsequent loss of independence, as well as a need for healthcare and social care at home (Feng et al., 2017). Nygren (Nygren, 2006) pointed out that concepts such as *healthy ageing, active ageing*, and *successful ageing* can describe good ageing, including physical, psychological, social, and spiritual dimensions. The older adults who participated in the current study focused on achieving and preserving good ageing through healthy, active, and successful lifestyles that included all the dimensions that they had highlighted as important. They had found their own ways to reach their goals, promoting and preserving their sense of health and well-being.

A balance in life—together with maintaining relationships and one’s own autonomy—were central components in the older adults’ lives, enabling them to uphold a sense of health and well-being. However, they did not wish to rely excessively on their children, grandchildren, or others because of the risk of becoming burdensome. This caution might have led them to withdraw from social interactions and spend more time by themselves. The older adults also emphasized the importance of maintaining relationships that made them feel well and avoiding relationships that they described as unhealthy. Such efforts to stay in contact with family members and friends can, according to Lundman et al. (Lundman et al., 2019), be regarded as an inner strength. Connectedness is one of four interacting cornerstones in the theoretical model by Lundman et al. (Lundman et al., 2010), described as the ability to make and keep friends, communicate well, and find meaning through involvement with other people, things, and one’s surroundings. The older adults who participated in the current study expressed a need to be useful, requested, and appreciated as a significant component of feeling well. This finding can be considered in light of research by Gruenwald et al. (Gruenewald et al., 2007) indicating that feeling useful is an often-unrecognized but significant predictor of older adults’ health and function. The other three interacting dimensions—creativity, firmness, and flexibility—in the theoretical model of inner strength by Lundman et al. (Lundman et al., 2010) also emerged among the current study’s findings. Participants were creative, firm, and flexible in finding ways to feel well despite illness and disease, interacting and maintaining healthy ageing. They coached themselves and strived to stay positive, focusing on opportunities instead of hindrances despite such adversities as vacillating health, anxiety, fears, and loneliness. They strived to remain firm, engaging in life and embracing opportunities to live meaningfully and feel well.

The older adults in the current study valued spending time with themselves and others, suggesting that they were comfortable on their own to some extent, without company, and did not feel alone. According to Courtin and Knapp (Courtin & Knapp, 2015), many older adults experience loneliness and social isolation, and maintaining relationships can, therefore, be difficult. These findings can be viewed in light of the ongoing global coronavirus pandemic and the research describing COVID-19 (coronavirus disease 2019) social distancing measures as major stressors for older adults, harming their well-being and causing a sense of loneliness (Macdonald & Hülür, 2021). Such considerations as the size of an individual’s social network, their frequency of social interaction, and their satisfaction with their social relationships have been described as connected to their subjective well-being and feelings of loneliness (Valtorta et al., 2016). A large social network (Bruine de Bruin et al., 2020), as well as more frequent social interaction (Amati et al., 2018), have been suggested to relate to higher levels of well-being.

Additionally, older adults can be understood in the current study as possessing the inner strength (Lundman et al., 2010) needed to meet such challenges as social isolation due to illness and disease and in situations as the pandemic Covid-19. The drivers of this inner strength can be understood in light of the current study’s findings. Besides upholding healthy relationships with family members and friends in order to maintain health and well-being, the older adults who participated in the current study prominently focused on and prioritized feeling well, as their perspectives, choices, and daily lifestyles explicitly revealed. They expressed changes as a natural part of life, largely viewing such transitions positively, and they were open to meeting challenges and changes in order to feel well. Furthermore, inner strength also encompasses the ability to engage in life and be open to embracing meaning in life, as well as flexibility, creativity, conscious decision-making—all of which emerged in this study’s results. Additionally, other aspects linked to inner strength—such as interactions with one’s self and others, enjoying nature, and allowing spiritual dimensions in life—were also observed. The older adults demonstrated the inner strength needed to embrace life and coach themselves towards healthy, meaningful lifestyles in order to promote and preserve their sense of health and well-being.

Identifying ways to maintain a healthy lifestyle and achieve a balance in life were central in promoting and preserving health and well-being after retirement. The older adults who participated in this study understood the importance of upholding healthy lifestyles both physically and psychologically, and they coached themselves to adopt such lifestyles accordingly. They balanced a daily commitment to healthy lifestyles with resting, and they conducted physical, cognitive, and social activities in order to feel well and remain healthy. This orientation prevailed among the older adults, helping them deal with uncomfortable thoughts, independently uphold habits and routines, and interact with and help others in order to uphold and improve their sense of health and well-being. The importance of striking such a balance in life has been highlighted in the process of healthy ageing (Gruenewald et al., 2007). The commitment to uphold such active lifestyles found in the current study has been acknowledged in earlier research. Passive lifestyles with sedentary behaviours (physically, such as sitting, and psychologically, such as using a computer) have been shown to influence health and well-being, including subjective well-being (Djernes, 2006; Kikuchi et al., 2014; Lampinen et al., 2006; Scott et al., 2020). Indeed, physical inactivity and inactive lifestyles have been shown to predict poor health and age-related infirmities (Dogra & Stathokostas, 2012; O’Neill & Dogra, 2016) and they have been associated, for example, with an increased risk of functional limitations affecting daily life (Gennuso et al., 2013), overweight, psychological distress (Kikuchi et al., 2014), and death (Rezende et al., 2014). Research has shown that behaviours before retirement are likely to continue into retired life. Healthy ageing has, therefore, become associated with non-sedentary, active lifestyles (Dogra & Stathokostas, 2012; O’Neill & Dogra, 2016; Scott et al., 2020; Ter Hoeve et al., 2020). Additionally, a sense of health and well-being has also been associated with meaning in life.

The current study’s findings have revealed the importance of feeling well in body and soul, and functioning independently in daily life in order to promote a sense of health and well-being. The ability to feel well and independently balance interactions, engagements, and activities also contributed to meaning in life and feeling young again. However, a threat to a sense of health and well-being, as well as overall healthy ageing, is the pervasive ageist perspective towards older adults in society at large. The older adults in this current study experienced these negative attitudes and expressed uncertainty and fear regarding prioritizing and access to healthcare when needed. Ageism is clearly a problem in today’s society and provision of healthcare, causing problems for older adults. Stereotypes and negative attitudes towards older adults are known to affect older adults’ sense of health and well-being (Burnes et al., 2019; Wyman et al., 2018), and they might directly contradict the development of older adults’ inner strength, hindering their sense of health and well-being.

The current study’s findings may be transferred to similar contexts (Sjöström & Dahlgren, 2002) involving older adults’ perceptions of health and well-being after retirement in Sweden. However, readers should note that this study’s participants were a group of older adults from only one region and two municipalities in Sweden. Furthermore, the proportions of women and men who participated in this research can be regarded as a limitation since fewer men than women participated. On the contrary, this variety between male and female participants can be regarded as mirroring current demographics in Sweden.

### Conclusion and implications

This study’s findings confirm that an absence of illness and disease is not necessary for older adults to achieve a sense of health and well-being. Additionally, healthy ageing emerged as a key focus in our interviews, suggesting—in accordance with the WHO (WHO, 2019)—that healthy ageing is not merely associated with physical aspects; rather, it also encompasses psychological and social aspects. The older adults’ lifestyles and embracing of life seemed to support the development of their inner strength. To promote and preserve health and well-being after retirement, these older adults strove for and coached themselves in upholding a balance in life, focusing on not burdening others. Their orientation towards living independently was grounded in their interactions with themselves and others, being useful, engaging in life, and maintaining a healthy lifestyle in order to achieve a sense of health and well-being. Their orientation towards post-retirement life must be acknowledged by society at large, especially from an ageist perspective, and in healthcare and social care in order to preserve and promote health and well-being. It is important to support older adults in their overall effort to feel well despite illness and disease in life after retirement while remaining at home. Gaining a deeper understanding of older adults’ sense of health and well-being is also important for designing healthcare and adequately satisfying these adults’ needs. Thus, insights from the perspective of older adults perceived as healthy are valuable. Such knowledge is also important to the education of professionals who will work with older adults across healthcare contexts. This knowledge is greatly important to society in general, but it is especially important for healthcare providers and retiree organizations in order to promote older adults’ health and well-being. Furthermore, this study’s findings suggest that inner strength and interactions between this phenomenon’s dimensions should be studied to fully capture and describe its meaning in life from the perspective of healthy older adults after retirement.
